# Editorial

**Published:** 1857-06

**Authors:** 


### Editorial.

### OUR EDITORIAL EXPERIENCE.

A year has elapsed, and this number closes a volume. A short experience, says one. But, hold on—we wish to do all the talking in this meeting, and, after it is adjourned, you can, if you like, hold one of your own, and, we'll not interrupt you. We didn't intimate that we have had a *long* experience; but, sometimes, we consider it a *strong* one.

Well, we set out full of hope and with high aspirations. You remember we spoke of enlarging the Register when you would give us the necessary aid in extending its circulation. And, really, we thought you would do that right off; and we, (that is *I;* for our "comrade" don't get excited) lay awake at night thinking of the "Dental Register, vol. xi. A quarterly journal, of 160 pages"—but there is no use in telling these symptoms now; for we have got entirely over that complaint.

You know—you ought to—that our terms are "in advance." With the second number, that is, at the middle of the year, we sent bills to those who had not remitted. We asked an experienced publisher how much we would be safe in expecting on those bills in a given time. His reply was, "About one-third of them will be paid in three weeks, and the half within a month." Rather encouraging, thought we. The result was that within the month, about *one subscriber in thirty* remitted.

We sent specimen numbers to some, with an urgent request that if they did not wish to be considered subscribers they would return them, or refuse them and let the postmaster notify us. Quite a number of new *paying* subscribers were thus obtained; and, in several instances, we were cordially thanked for sending the specimen. Some kept three numbers and notified us to discontinue. A few kept the three numbers, refused to pay for them, and wrote impertinent, saucy letters, besides. They took the goods at the marked price, and on the published terms; and, had the article been *cloth* instead of *paper*, they would know as well as any one that they are guilty of fraud in refusing to pay for it. But there are not many such men in the profession.

With a subscription list that would make us feel rich if each one would only send that "three dollars in advance," we have *already* received about half as much as the cost of issuing. Well, do you think we are discouraged? Nothing of the kind, gentlemen. Our hopes are not so buoyant as they were a year ago; but they are better balanced. We know that the Register has *some* friends wide awake now; and when we see people enjoying a very comfortable sleep, we naturally expect great energy when they awake; and, hence, we know that very many of you who have not remitted for vol. x, will soon send along *six*, instead of three dollars, and obtain your receipts for vols x. and xi. at the same time. We know, also, that many of you who seldom write, and some who never do, will send practical articles for our pages, making the original department of the Register more extensive than that of any other dental periodical. We know too, that the Register is a "fixed fact," and will live, even if we die at our posts.

We trust that we have learned something by even a short experience. If so, we know better how to work; and we do not expect either to rest or play. And while we are willing to work, we also expect you to help us.

Now, gentlemen, send on your communications, and in good time. Don't say you can't write; for you can. One of you, who still maintains he can do nothing with the pen, has, at last, by long teasing, sent us a highly instructive and practical article, but too late for this number. Better late than never, though.

One more item of our experience, and we are done. We find the more we labor for, the more warmly we become attached to the profession. No labor is to us more pleasant than that bestowed on the Register; and we trust that, through your co-operation and counsel, this labor will be properly directed.

---

☞ By a miscalculation of our space and matter, a number of editorial notices are crowded out. We hope all concerned will have patience. We delayed this issue to obtain the proceedings of the Western Dental Society; and we are sure our readers will excuse the delay.

---

### LESLIE'S ADHESIVE FOIL.

The adhesive properties of this foil are unsurpassed. This is true both of James Leslie's, and of A. M. Leslie's. Those present at the meeting of the Mississippi Valley Association may feel curious to hear from the three leaves of foil which Mr. Leslie annealed and then *welded* together by the pressure of his finger. Well, they are still, in the strictest sense, *one leaf.* And the ring made from A. M. Leslie's foil, and presented by him to his *Alma Mater*, the Ohio College, is still as solid as if cast in an ingot. We mention these things, in part, for the consolation of those who fear that gold thus *welded* will some day fall apart, if not turn to "brick dust."

### WESTERN DENTAL SOCIETY.

We had the pleasure of being present at, and witnessing the proceedings, and hearing the discussions of this association, which met in St. Louis on the 20th of May.

Its sessions occupied three days and two evenings. The work was entered into with a hearty good will, and a determination to do something; and it was done. They met at 9, A. M., and worked till 1 or 2; met at 3, and worked till 6; met at 8, and worked till 10, P. M.

The time was not spent in fruitless janglings and contentions. The machinery was immediately set in motion by the president, Dr. Edward Hale, sen. Under his administration it moved with beauty and grace. He was, after a time, succeeded by Dr. Isaiah Forbes. His order was, put on the steam.

The principles and practices of the profession were taken hold of, examined, investigated and discussed, with a thoroughness seldom equalled in such bodies. There were not a great number of subjects for discussion assigned, in the order of business; but, upon each of these, there was a free expression of opinion, and many new particulars in practice were elicited, a budget of which, doubtless each one took home with him. We can vouch for the memorandum of one, which shows sundry scribbles indicating new ideas.

Besides the regular order, there were various topics incidentally introduced, all of which excited much interest.

There was one feature introduced which we have not observed elsewhere. Each member was requested to confine his remarks to a particular case or point; for instance, upon the subject of filling teeth, each member in his remarks confined himself to some particular cavity, describing its location, extent, and character—then describing minutely every step in his method of filling that cavity. In this way, no ground was twice gone over; as is frequently the case in random discussions. We have no particular objection to going twice over the ground, if sub-soiling is needed; but then it would be better to run the furrows the same way, and not across one another.

The members did not become weary—the interest did not flag; but increased from the beginning to the end.

The attendance was good—most of the Western states were represented.

Quite a number of new members were added to the society, and from the length of time occupied by the examining committee with the candidates, and from their appearance after they run through, we rather surmised that they thought the way into that society was a "hard road to travel."

Gentlemen of the profession, unless you are about right, in that which constitutes the *man*—in integrity and intentions, and also professional attainments, you need not "*knock at the door*" of the "Western Dental Society for admission.

Every man who witnessed the proceedings would go home a better man professionally, and with a firm resolve to do something for the elevation of the profession. T.

### AMERICAN DENTAL CONVENTION.

The Corresponding Secretary's notice of the next meeting of the Convention was intended for the March number, but was not received in time. We hope the West and South will be fully represented. We are sorry that we have not the report of the "Business Committee," for our present number, but we are not disappointed; for large committees, especially when *diffused*, are not prompt. If the order of business is not published beforehand, we will have to content ourselves with another general off-hand fire.

### ANOTHER INSTRUMENT FOR DRYING CAVITIES.

Dr. Leslie, of St. Louis, exhibited to the society recently held in that place, an instrument for drying cavities in teeth, and particularly useful for drying those partially filled, that have become moistened during the operation. It consists simply of a burr drill, of the proper size—different sizes may be employed—over which fibres of asbestos are doubled, passing a little way beyond the bulb on the shaft of the instrument. Here it is bound firmly on with fine platinum wire, and the instrument is now ready for use.

Place it in the flame of a spirit lamp, a moment or two, or till the bulb becomes as warm as is desired, then apply to the cavity: Previous to this, however, wipe out with cotton, thus removing as much of the moisture as possible. Then apply the dryer, which renders the cavity entirely free from moisture. The bulb retains the heat and dries the moisture from the asbestos. It can be re-warmed, if necessary. It will accomplish the work almost as well as the warm air blowpipe; but we want them both. Thanks to the man with whom it originated. Will somebody tell us who?

### SATTERTHWAITE'S TEETH.

We have seen some beautiful specimens of teeth, manufactured by Dr. Satterthwaite, expressly to be used in his style of porcelain work. They are without rivets or metallic attachments of any kind; and, in respect of color, form, and texture, they are emphatically up with the times. Dr. S. is one of the early graduates of Ohio College; and his "specimen set" still stands in the cabinet, displaying teeth manufactured by himself at that early day. He was up with the times then, and he seems but little inclined to fall behind. We were unable to send you a specimen of those French teeth, Doctor.

☞ In sending bills with the last number some mistakes occurred. That is, some received bills not their own. This occurred in the mailing, which is not done by ourselves. If any who owe us received other men's bills, we hope they will not be too bashful to remit without waiting for their own. Any who do not owe us will please excuse the mistake. And now, gentlemen, don't conclude, that because we send no bills with this number, we do not need money. The printers must still be paid. If you take the Register and have not paid for it, you know that you owe us, as well without a bill as with it; and we hope you will remit by mail, without delay, at our risk. To all who have aided us with either money or their pens, we return our warmest thanks; and, especially to the few who have already advanced on vol. XI.

### THE DENTAL RECORDER.

Too late to be noticed in our March number we received the following, which fully explains itself:

"To THE PATRONS OF THE DENTAL RECORDER.—The 10th Volume of this Journal ends with the present number.

With an ample Subscription List, we find our *collections* inadequate to paying the expenses of the Magazine: we have therefore concluded for the future, to avail ourselves, more than heretofore, of its value as an advertising medium, and to issue numbers, only at such intervals, as may suit our convenience.

To subscribers who have assisted in sustaining our Magazine thus far, we tender our sincere thanks.

FEB. 28, 1857. SUTTON & RAYNOR."

Well, we are sorry—and fear that our sorrow is as that of those *who have no hope.* How truly could we erase "Sutton & Raynor" and subscribe the name of our own firm, were it not for the latter clause of the middle sentence. "The 10th Volume of THIS Journal ends with the present number." "With an ample subscription list, &c." True with us also. "We have therefore concluded, &c." Alas! no. We have *nothing* to advertise. Happy fellows! If the profession won't pay for a Journal they will buy your goods: for they can make money by so doing.

But, if the "Recorder" has, to a certain extent retired from the contest, by looking at the advertising sheet of the "Register," it will be seen that "SUTTON & RAYNOR" are still on hand, and in full blast, with every thing that the professional man desires or needs. Send on your orders and try them.

### PLASTER CASTS.

With plaster which is coarse in quality or badly calcined, it is often difficult to obtain perfectly smooth models. We have recently witnessed some highly satisfactory results, with very *unsatisfactory* plaster, in the hands of Dr. E. B. Loud. His method is this: Take an excess of water, add the plaster gradually to it, allowing it to sink and thus become thoroughly saturated. Then pour off the excess of water and use before the plaster is too much set. The fear that some others may be annoyed as we have been is our only apology to Dr. L. for thus bringing his name, unexpectedly to him, into our pages. W.

